# SRC as a Prognostic and Immunomodulatory Biomarker in Acute Myeloid Leukemia: A Multi-Omics Study

**DOI:** 10.3390/ijms27093734

**Published:** 2026-04-22

**Authors:** Jirui Zhong, Xikun Liu, Xuekui Gu, Zenghui Liu

**Affiliations:** 1 The First Clinical Medical School, Guangzhou University of Chinese Medicine, Guangzhou 510405, China; 2 Department of Hematology, The First Affiliated Hospital of Guangzhou University of Chinese Medicine, Guangzhou 510405, China

**Keywords:** acute myeloid leukemia, SRC, tumor microenvironment, immunosuppression, prognosis, bioinformatics

## Abstract

The bone marrow tumor microenvironment (TME) is critical for acute myeloid leukemia (AML) progression, immune evasion, and treatment resistance. SRC, a non-receptor tyrosine kinase involved in multiple oncogenic pathways, has not been systematically characterized in AML in relation to prognosis and immune regulation. We integrated bulk transcriptomic and single-cell RNA-sequencing datasets from TCGA, BeatAML, and GEO. Immune-related targets were identified using xCell-based immune scoring and weighted gene co-expression network analysis (WGCNA), followed by protein–protein interaction analysis and multi-algorithm machine-learning screening. We then evaluated SRC expression patterns, prognostic associations, immune microenvironment features, predicted drug sensitivity, single-cell differentiation dynamics, intercellular communication, and in silico virtual knockout perturbation (scTenifoldKnk). SRC emerged as the most robust hub gene after integration of WGCNA, PPI analysis, machine-learning feature selection, and survival screening. SRC was significantly upregulated in AML compared with normal controls and was independently associated with poor overall survival (HR = 1.231, *p* = 0.037). High SRC expression was linked to adverse ELN/FAB features, increased immune checkpoint expression, enrichment of inflammatory and immunoregulatory pathways, and a higher proportion of primitive leukemia-associated cell states. Single-cell analyses further suggested that SRC was enriched in CD34^+^ progenitor compartments, associated with altered cell–cell communication, and accompanied by distinct mutation and pathway profiles. Drug-response prediction and in silico network perturbation analysis further supported the potential biological and translational relevance of SRC-centered programs. SRC is a prognostically relevant and immune-associated hub linked to AML microenvironment remodeling, and may serve as a candidate biomarker and potential therapeutic target that warrants further experimental validation.

## 1. Introduction

Acute myeloid leukemia (AML) is a malignant clonal disorder arising from hematopoietic stem/progenitor cells in the bone marrow. It is characterized by uncontrolled proliferation, differentiation arrest, and impaired apoptosis of myeloid precursor cells [1]. These abnormalities lead to the accumulation of blasts and immature cells in the bone marrow and peripheral blood, resulting in severe complications such as anemia, infection, and bleeding [2]. AML is the most common acute leukemia in adults, and its incidence increases markedly with age, posing a persistent challenge for the clinical management of hematologic malignancies [3,4]. In recent decades, substantial progress in understanding the molecular basis of AML has promoted the development of targeted and epigenetic therapies, including FLT3 inhibitors [5,6], IDH1/2 inhibitors [7], BCL-2 inhibitors (e.g., venetoclax) [8,9], and hypomethylating agents [10]. Nevertheless, long-term survival remains unsatisfactory, particularly in elderly patients and those with relapsed/refractory disease, who generally have poor outcomes [11,12]. The marked genetic heterogeneity of AML and its adaptive interactions with the tumor microenvironment (TME) are considered major drivers of treatment resistance and relapse [13,14].

In recent years, research has increasingly shifted from leukemia cell-intrinsic genetic abnormalities to the microenvironment in which leukemic cells reside, namely the tumor microenvironment (TME) [15,16]. The AML TME is a dynamic and complex ecosystem composed of bone marrow mesenchymal stromal cells, endothelial cells, osteoblasts, adipocytes, diverse immune cell populations, and acellular components [17]. Accumulating evidence indicates that the TME is not a passive bystander; rather, it actively contributes to AML initiation, progression, immune evasion, and treatment resistance [18,19]. Through bidirectional signaling crosstalk, leukemic cells and stromal/immune components co-establish a permissive niche characterized by immunosuppressive features, pro-survival signaling, pro-angiogenic activity, and support for leukemia stem cells (LSCs) [20,21]. LSCs are a major source of relapse and drug resistance in AML. These cells often reside in specialized bone marrow niches and maintain a relatively quiescent state, which helps them evade the cytotoxic effects of conventional chemotherapy [22,23]. Therefore, a deeper understanding of the cellular and molecular architecture of the AML TME, as well as the key mediators of leukemia-microenvironment crosstalk, is essential for developing strategies to eradicate LSCs, reverse immunosuppression, and overcome drug resistance.

SRC, the first identified cellular proto-oncogene, encodes a non-receptor tyrosine kinase belonging to the Src family kinases (SFKs). As a broadly utilized signaling molecule, SRC functions downstream of multiple receptor systems, including growth factor receptors, cytokine-related pathways, and adhesion-associated signaling complexes, thereby integrating extracellular cues with intracellular programs that regulate cell proliferation, survival, adhesion, migration, and cytoskeletal remodeling [24,25]. In cancer biology, aberrant SRC expression or activation has been widely implicated in tumor initiation and progression through promotion of oncogenic signaling, invasive behavior, epithelial–mesenchymal transition-like phenotypes, angiogenesis, and therapeutic resistance. In solid tumors, SRC promotes tumor progression and metastasis through multiple mechanisms, including integration of receptor-mediated signaling (e.g., EGFR and PDGFR pathways) [26,27], modulation of focal adhesion kinase (FAK) activity [28], disruption of cell–cell junctions (e.g., E-cadherin) [29], and induction of vascular endothelial growth factor (VEGF) expression [30]. However, the role of SRC in AML remains incompletely understood. Previous studies suggest that SRC may participate in downstream signaling of oncogenic mutations such as FLT3-ITD [31,32,33], but its clinical relevance, prognostic value, and potential functions in immune regulation, intercellular communication, and bone marrow niche remodeling in AML are still unclear. Notably, emerging evidence also supports the therapeutic potential of targeting SRC-related pathways in AML. For example, bosutinib (SKI-606) has been reported to enhance the anti-leukemic activity of MCL-1 inhibitors by promoting ubiquitin-mediated degradation of MCL-1 [34], while the myristoylation inhibitor zelenirstat induces endoplasmic reticulum stress and apoptosis and reduces leukemia burden in AML models, with notable activity in leukemia stem cell-enriched populations [35]. Together, these findings underscore the need for a systematic evaluation of SRC in AML, particularly its role within the bone marrow immune microenvironment.

Against this background, the present study aimed to systematically investigate the regulatory role of SRC in the AML immune microenvironment using an integrative multi-omics framework. We combined bulk transcriptomic and single-cell RNA-sequencing data with weighted gene co-expression network analysis (WGCNA), protein–protein interaction network analysis, and multiple machine-learning algorithms to identify core immune-related hub genes, and SRC was selected for in-depth investigation.

We then performed a multi-dimensional analysis centered on SRC, including its expression pattern in AML, associations with clinical features and prognosis, effects on immune microenvironment remodeling (immune cell infiltration, immune checkpoint expression, and pathway activity), and its dynamic roles in cellular differentiation, state transitions, and intercellular communication at single-cell resolution. In addition, we explored the association between SRC expression and predicted drug sensitivity and conducted an in silico perturbation analysis to infer downstream network effects of SRC disruption. Collectively, this study provides a comprehensive view of the potential role of SRC in AML progression and immune microenvironment remodeling, and supports its candidacy as a prognostic biomarker and potential therapeutic target.

## 2. Results

### 2.1. WGCNA Identifies Immune-Related AML Targets (IRATs)

**Figure 1:**
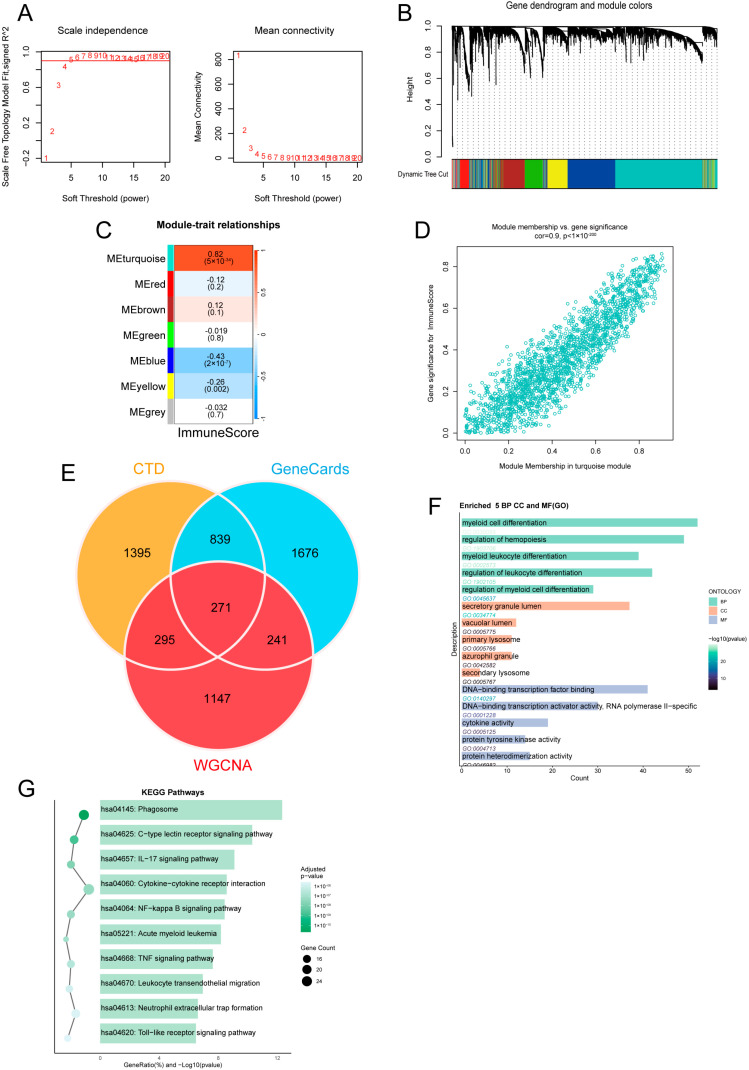
Identification of Immune-Related AML Targets (IRATs) via Weighted Gene Co-expression Network Analysis (WGCNA). (**A**) Soft-threshold power selection. (**B**) Gene clustering dendrogram (1-TOM). (**C**) Co-expression modules identified by WGCNA. (**D**) Correlation between module membership (MM)and gene significance (GS) for ImmuneScore in the turquoise module. (**E**) Venn diagram of overlapping genes. (**F**) Enriched KEGG pathways. (**G**) Enriched GO terms (BP, CC, MF). AML, Acute Myeloid Leukemia. IRATs, Immune-Related AML Targets.

Based on xCell-derived immune scores in the TCGA-LAML cohort, WGCNA was used to construct a scale-free gene co-expression network (Figure 1A). Multiple co-expression modules were identified using dynamic tree cutting (Figure 1B,C). Correlation analysis showed that the turquoise module exhibited the strongest association with ImmuneScore (Figure 1D) and was therefore selected as the core immune-related module. Intersecting genes from this module with AML-related targets from the CTD and GeneCards databases yielded 271 immune-related AML targets (IRATs) (Figure 1E). Functional enrichment analysis demonstrated that these IRATs were significantly enriched in pathways related to the phagosome, C-type lectin receptor signaling, IL-17 signaling, and acute myeloid leukemia (Figure 1F), as well as biological processes associated with hematopoietic cell differentiation and immune cell differentiation (Figure 1G). These results suggest that the identified IRATs are closely involved in AML pathogenesis and immune regulation.

### 2.2. Core Gene Identification

**Figure 2:**
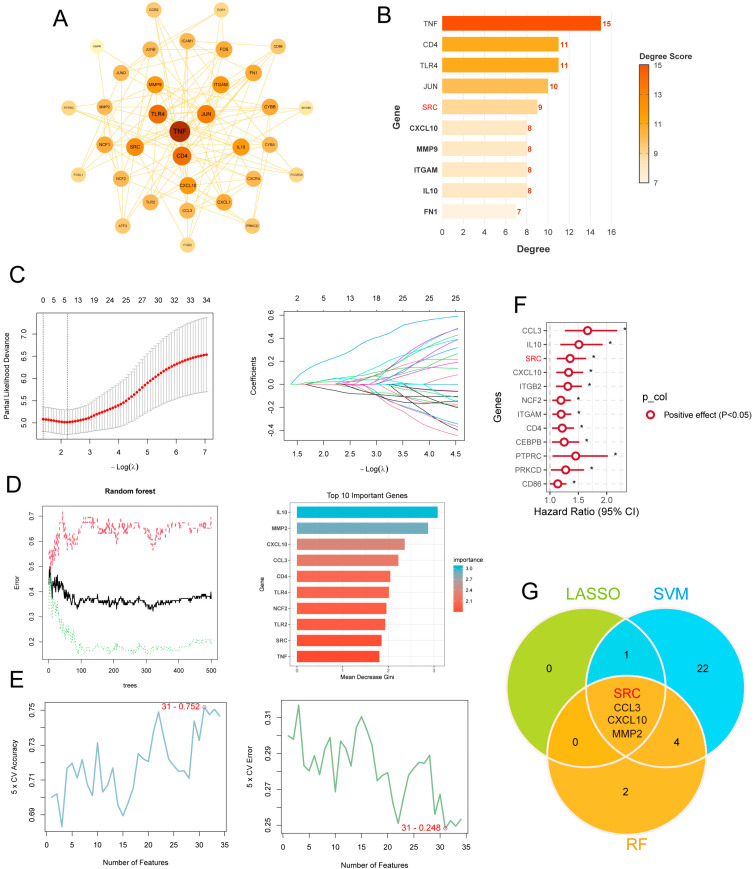
Identification of SRC as a Core Immune-Related Gene in AML through Machine Learning Integration. (**A**) Protein–protein interaction (PPI) network of core immune genes. Node color and size represent degree score. (**B**) Top 10 hub genes by degree. (**C**) LASSO regression analysis for feature selection. **Left**: Partial likelihood deviance plotted against −log(λ), with the red dotted line indicating the optimal λ value selected by 10-fold cross-validation. **Right**: LASSO coefficient profiles of the candidate genes, where each colored line represents a single gene’s coefficient trajectory as λ varies. (**D**) Random forest analysis showing gene importance. (**E**) Feature selection via SVM-RFE analysis. **Left**: Error rate curves for the random forest model, with black, red, and green lines representing overall error, positive class error, and negative class error, respectively, plotted against the number of decision trees. **Right**: Top 10 important genes ranked by Mean Decrease Gini. (**F**) Prognostic assessment by univariate Cox regression. (**G**) Venn diagram identifying SRC, CCL3, CXCL10, and MMP2 as consensus core genes. * *p* < 0.05.

To identify central hub genes among the IRATs, we constructed a protein–protein interaction (PPI) network (Figure 2A) and ranked candidate hub genes using the Cytoscape cytoHubba plugin (Cytoscape v3.9.1). Genes including SRC and CXCL10 consistently ranked highly across multiple topological algorithms, indicating central positions in the network (Figure 2B). We next performed secondary feature selection using three machine-learning methods: LASSO regression (Figure 2C), Random Forest (Figure 2D), and SVM-RFE (Figure 2E). In parallel, univariate Cox regression identified 12 genes significantly associated with poor prognosis (Figure 2F). Intersecting the results across network analysis, machine-learning filters, and survival analysis yielded four consensus core genes: SRC, CCL3, CXCL10, and MMP2 (Figure 2G). Given its prominent network centrality, frequent selection across algorithms, and prognostic relevance, SRC was selected as the core target gene for subsequent analyses.

### 2.3. Clinical and Prognostic Significance of SRC

**Figure 3:**
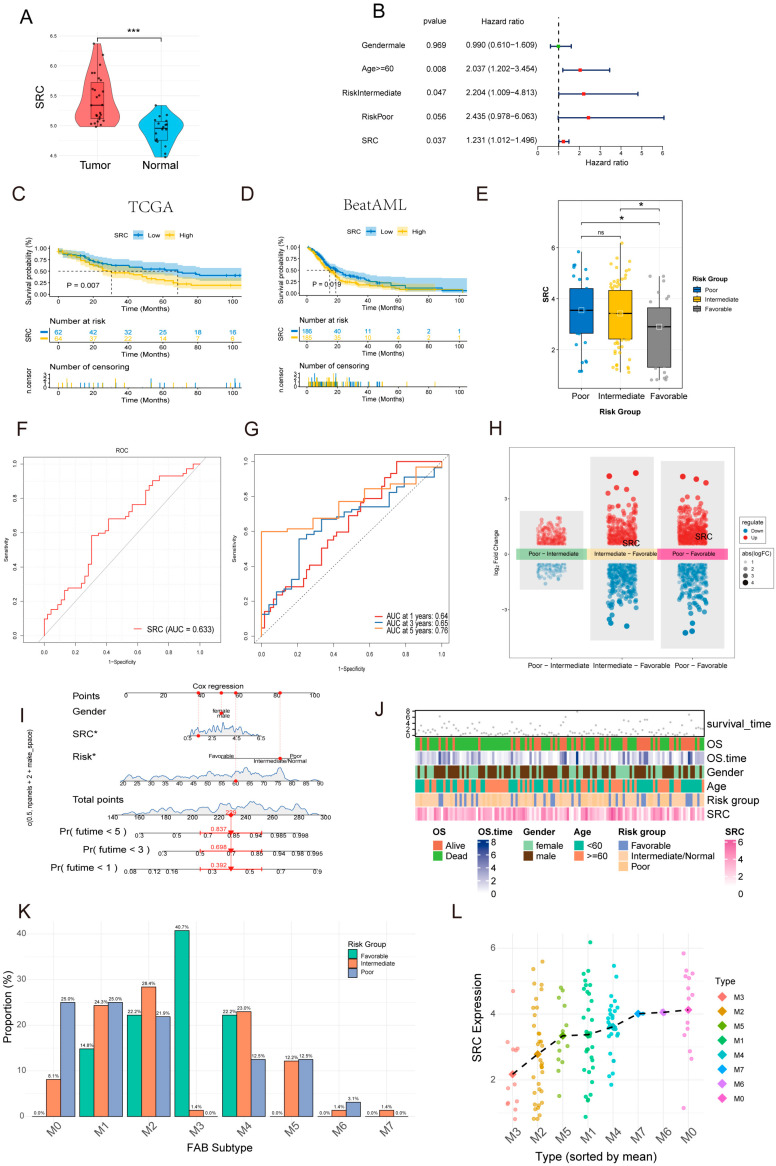
The Clinical and Prognostic Significance of SRC in AML. (**A**) SRC is significantly upregulated in AML vs. normal samples. (**B**) Multivariate analysis confirming independent prognostic value. (**C**,**D**) High SRC expression correlates with worse overall survival in TCGA and BeatAML cohorts. (**E**) SRC expression is highest in the ELN poor-risk group. (**F**,**G**) Predictive performance of the SRC signature. (**H**) Differential expression between risk groups. (**I**) A nomogram integrates SRC with clinical variables for predicting survival probabilities. (**J**) Gene expression-clinical trait association heatmap. (**K**) Bar plot showing the proportion of ELN risk groups (favorable, intermediate, poor) within each FAB subtype. (**L**) SRC expression is highest in FAB M0 subtypes. ns, not significant; * *p* < 0.05; *** *p* < 0.001.

We first confirmed that SRC expression was significantly higher in AML samples than in normal controls (Figure 3A). In multivariable Cox regression analysis adjusted for clinical confounders (including age and risk category), SRC remained an independent prognostic risk factor (HR = 1.231, *p* = 0.037; Figure 3B). Notably, gender, age, risk stratification, and FAB classification were also significantly associated with prognosis in the model.

Kaplan–Meier survival analysis showed that patients with high SRC expression had significantly shorter overall survival in both the TCGA-LAML cohort and the independent BeatAML cohort (Figure 3C,D). SRC expression also showed a significant positive association with ELN risk stratification, increasing progressively from lower- to higher-risk groups (Figure 3E). The AUC of SRC as a single prognostic factor was 0.633, and time-dependent ROC analysis yielded AUC values of 0.64, 0.65, and 0.76 for predicting 1-, 3-, and 5-year survival, respectively (Figure 3F,G), indicating moderate prognostic discrimination that was more pronounced for longer-term survival.

Across cytogenetic risk subgroups, SRC expression was elevated in the intermediate- and high-risk groups (Figure 3H). A nomogram integrating SRC expression with clinical variables showed good utility for predicting 1-, 3-, and 5-year survival probabilities (Figure 3I). Consistently, the gene expression–clinical trait heatmap indicated that higher SRC expression was associated with adverse clinical features and poorer prognosis (Figure 3J).

In addition, ELN risk distribution varied across FAB subtypes (Figure 3K): the M3 subtype was predominantly low-risk, whereas M0, M5, M6, and M7 subtypes were represented mainly in the intermediate- and high-risk groups. SRC expression also differed significantly among FAB subtypes, with the lowest expression in the prognostically favorable M3 subtype and the highest expression in the poorest-prognosis M0 subtype (Figure 3L). Collectively, these findings support SRC as a clinically relevant biomarker associated with AML progression and risk stratification.

### 2.4. SRC Is Associated with an Immunosuppressive Microenvironment

**Figure 4:**
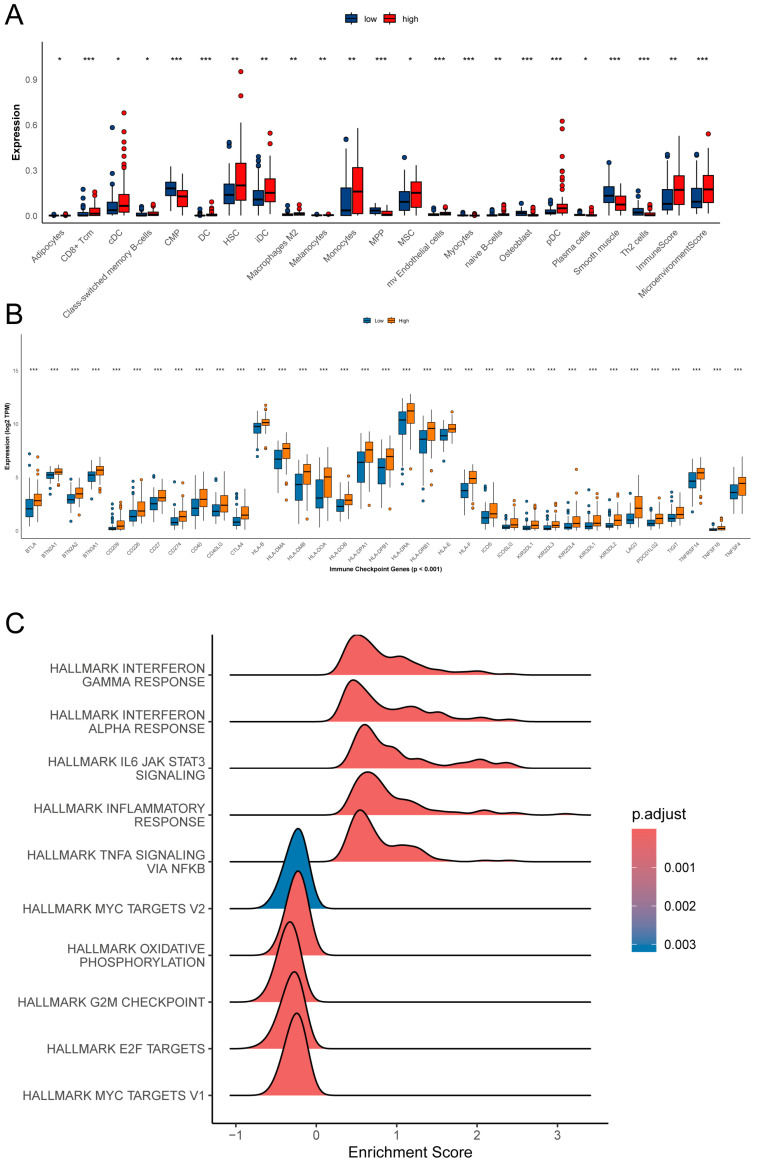
Exploring the functional implications of SRC expression. (**A**) Tumor immune microenvironment landscape stratified by SRC expression. (**B**) Differential expression of immune checkpoints. (**C**) GSEA reveals enriched biological pathways. not significant; * *p* < 0.05; ** *p* < 0.01; *** *p* < 0.001.

After stratifying samples into SRC-high and SRC-low groups using the median SRC expression, xCell-based immune infiltration analysis showed that the SRC-high group was significantly enriched in immunosuppressive cell populations, including M2 macrophages and plasma cells, and had higher overall ImmuneScores (Figure 4A). In parallel, the SRC-high group exhibited significantly increased expression of multiple immune checkpoint molecules, including CD274 (PD-L1), CTLA4, LAG3, and TIGIT (Figure 4B).

Gene set enrichment analysis (GSEA) further indicated that the SRC-high state was enriched in inflammatory and immune-related pathways, including TNF-α/NF-κB signaling, inflammatory response, IL6-JAK-STAT3 signaling, and interferon response pathways (Figure 4C). By contrast, the SRC-low group was enriched in cell cycle-related pathways. These results suggest that high SRC expression is associated with an AML phenotype characterized by immune/microenvironmental remodeling rather than predominantly proliferative signaling.

### 2.5. Drug Sensitivity Analysis

**Figure 5:**
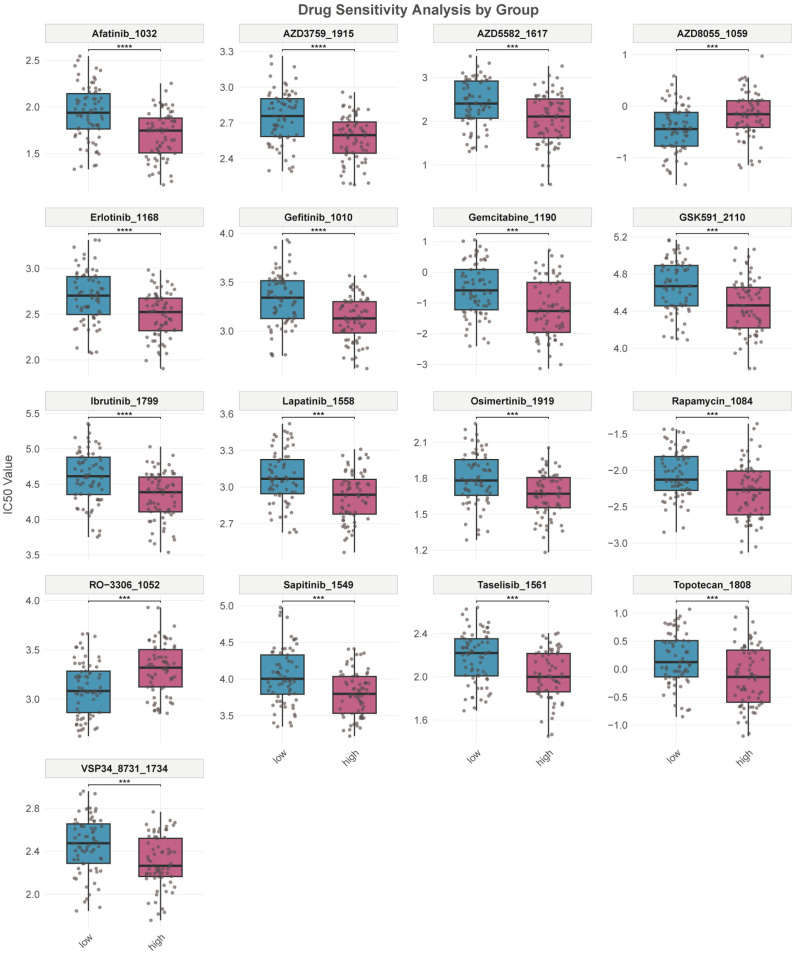
SRC expression reveals differential drug sensitivity. Comparison of estimated IC_50_ values between SRC-high and SRC-low AML groups for a panel of compounds. Blue boxes represent the SRC-low group, and red boxes represent the SRC-high group. *** *p* < 0.001; **** *p* < 0.001.

Transcriptome-based drug sensitivity prediction showed that the estimated IC50 values of several compounds, including afatinib, ibrutinib, lapatinib, and gemcitabine, were significantly lower in the SRC-high group than in the SRC-low group (Figure 5). These findings suggest that SRC-high AML may exhibit distinct predicted drug-response patterns. However, because these compounds are not standard therapies for AML, these findings should be interpreted cautiously and regarded as hypothesis-generating only.

### 2.6. SRC Expression Patterns in AML Hematopoietic Hierarchy

**Figure 6:**
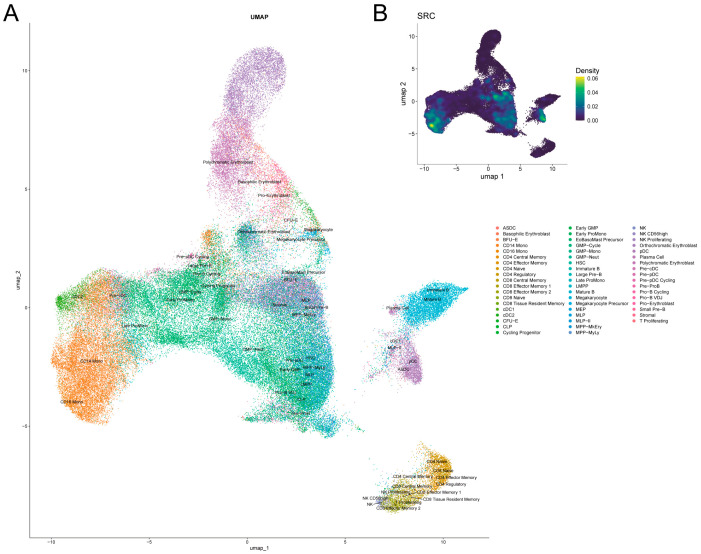
Single-cell mapping of hematopoietic differentiation and SRC expression. (**A**) UMAP projection annotating 55 distinct cell states. (**B**) Characterization of SRC Expression Across the Cellular Hierarchy.

To characterize SRC expression within the AML bone marrow microenvironment at single-cell resolution, we performed clustering analysis using the Seurat pipeline. UMAP visualization identified 55 distinct cell states (Figure 6A), spanning the hematopoietic hierarchy from hematopoietic stem/progenitor cells (HSPCs) to terminally differentiated cells of multiple lineages. Further analysis revealed marked heterogeneity in SRC expression across cell subpopulations. SRC expression was significantly elevated in CD34^+^ hematopoietic stem/progenitor cells (HSCs/MPPs), monocytes, and plasmacytoid dendritic cells (pDCs) (Figure 6B), suggesting that SRC may be functionally relevant in these cell compartments within the AML microenvironment.

### 2.7. SRC Is Associated with Hematopoietic Differentiation Dynamics

**Figure 7:**
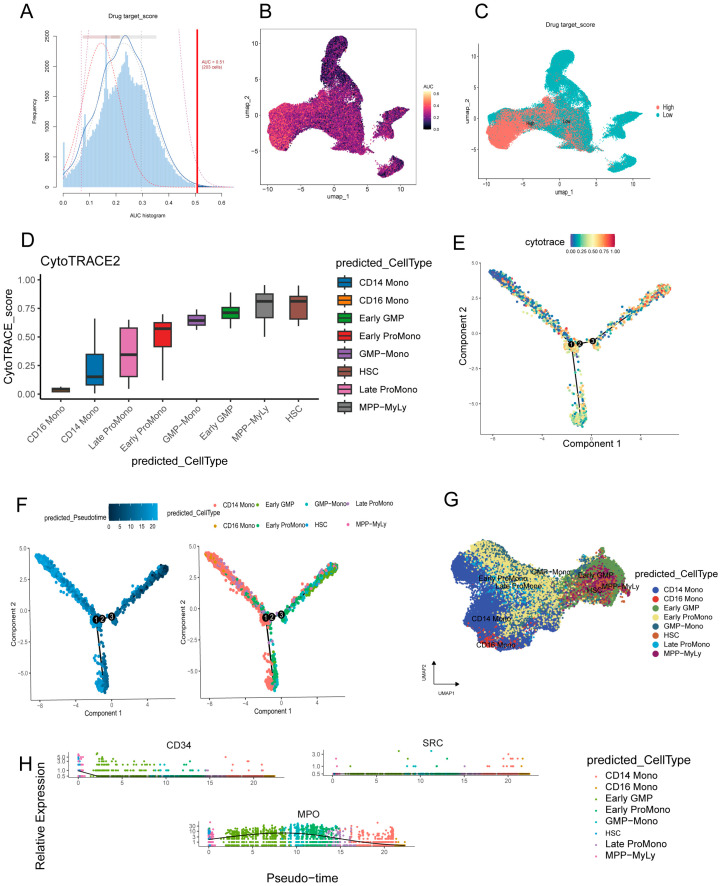
Regulatory dynamics and lineage commitment in hematopoiesis. (**A**) Histogram of hub gene activity scores. (**B**,**C**) Spatial visualization of gene regulatory activity on UMAP. (**D**) Cellular stemness prediction. (**E**) Cell state topology. Numbers 1, 2, and 3 mark key branching points in the hematopoietic differentiation trajectory, representing sequential lineage commitment decisions. (**F**) Reconstructed pseudotemporal progression. (**G**) Phylogenetic structure of monocytic lineage. (**H**) Expression kinetics of SRC and marker genes (CD34, MPO) along differentiation.

To further explore the role of SRC in cell-state transitions and differentiation trajectories in AML, we performed pseudotime analysis on the single-cell dataset. As shown in Figure 7, we first quantified SRC regulatory activity across cell states (Figure 7A) and visualized its distribution in UMAP space (Figure 7B,C). We then reconstructed the hematopoietic differentiation trajectory by integrating stemness estimation (Figure 7D), cell-state topology analysis (Figure 7E), and pseudotemporal ordering (Figure 7F), and further delineated the phylogenetic structure of the monocytic lineage (Figure 7G).

Along the inferred differentiation trajectory, we tracked SRC expression together with canonical hematopoietic markers, including CD34 (primitive-state marker) and MPO (myeloid differentiation marker) (Figure 7H). SRC expression remained relatively stable during progression from HSCs/MPPs to monocytes, whereas CD34 decreased progressively and MPO peaked at the GMP stage. These findings suggest that SRC may function as a broadly active signaling node associated with signal transduction across primitive and transitional myeloid states, rather than as a stage-specific differentiation marker. The relatively persistent expression of SRC along the inferred trajectory may therefore reflect preserved signaling competence across multiple cellular states in AML, rather than AML-specific lineage-restricted regulation.

### 2.8. SRC Is Associated with Altered Cellular Communication in the AML Microenvironment

**Figure 8:**
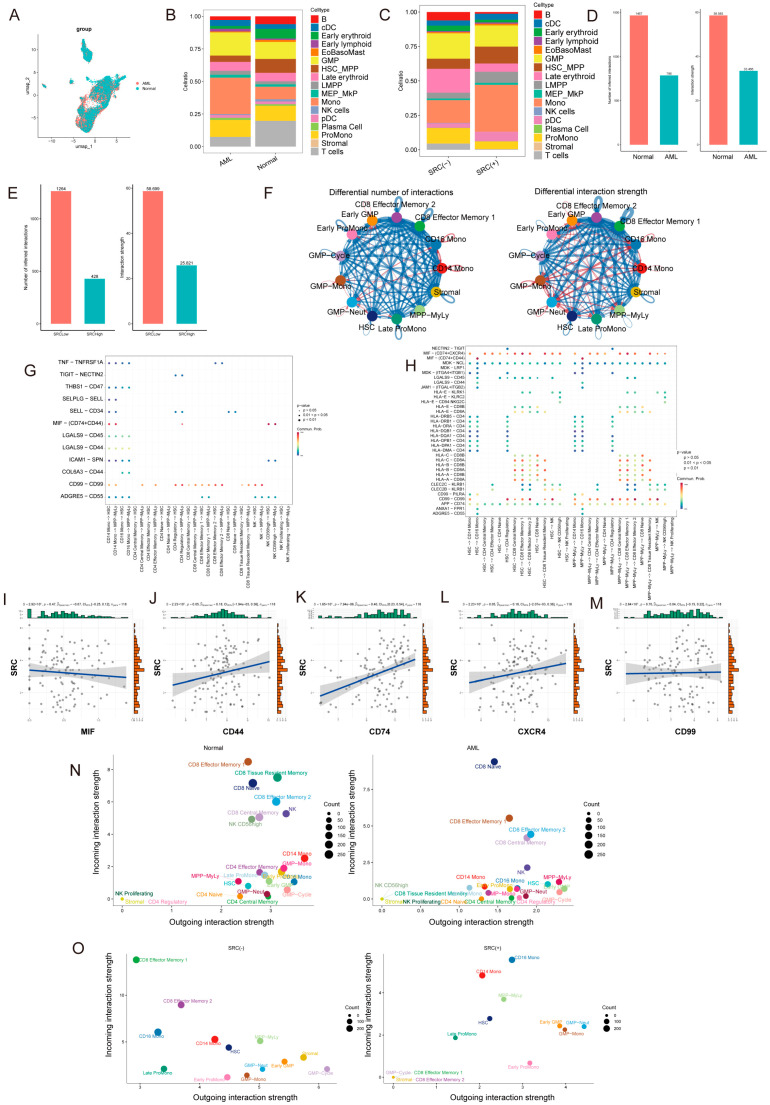
Single-cell atlas reveals cellular composition and communication differences among AML, normal, and SRC-expressing subgroups. (**A**) UMAP visualization of normal and AML samples. (**B**,**C**) Cellular composition in (**B**) AML vs. normal, and (**C**) SRC(+) vs. SRC(−) AML. (**D**–**F**) Global cell–cell communication: (**D**) AML vs. normal; (**E**,**F**) SRC(+) vs. SRC(−). (**G**,**H**) Significantly upregulated interactions along the immune cell–leukemia stem cell axis. (**I**–**M**) Correlation between SRC expression and key receptor signaling activity. (**N**,**O**) Functional shift in signaling roles: (**N**) Comparison of cell–cell communication patterns between AML and normal samples. (**O**) Comparison of cell–cell communication patterns between SRC(+) and SRC(−) group.

To investigate whether SRC expression is associated with changes in intercellular communication within the AML microenvironment, we performed a systematic single-cell communication analysis. UMAP visualization showed clear transcriptomic separation between AML and normal bone marrow cells (Figure 8A). Composition analysis further indicated that AML samples contained reduced proportions of HSCs/MPPs, T cells, and NK cells, but an increased proportion of monocytes, relative to normal bone marrow (Figure 8B).

When AML samples were stratified by SRC expression, the SRC-high group exhibited significantly higher proportions of HSCs/MPPs, monocytes, and pDCs than the SRC-low group (Figure 8C). Global cell–cell communication analysis showed that both the overall AML group and the SRC-high subgroup displayed a reduced total number of intercellular interactions compared with normal bone marrow or SRC-low AML, respectively (Figure 8D–F), suggesting a communication-suppressed immune microenvironment.

Despite the global reduction in interaction counts, specific signaling pathways linking HSCs/MPPs and immune cells were selectively enhanced in AML, including MIF-(CD74 + CXCR4), MIF-(CD74 + CD44), and CD99-CD99 signaling (Figure 8G,H). Moreover, SRC expression was positively correlated with expression of key receptors in these pathways (CD74, CXCR4, and CD44; Figure 8I–M), supporting an association between SRC and activation of these signaling axes.

Functional communication pattern analysis further suggested immunosuppressive network features in AML, with HSCs/MPPs and GMPs acting as major outgoing signal senders (Figure 8N). Notably, after stratifying AML samples by SRC expression, we observed a communication reprogramming pattern in the SRC-high group: although HSCs/MPPs and GMPs remained dominant signal sources, their principal signal recipients shifted from CD8^+^ T cells toward monocytes (Figure 8O). This finding suggests that high SRC expression is associated with redistribution of leukemia-centered signaling toward myeloid compartments.

### 2.9. SRC Expression and Leukemia Cell Differentiation and Prognosis

**Figure 9:**
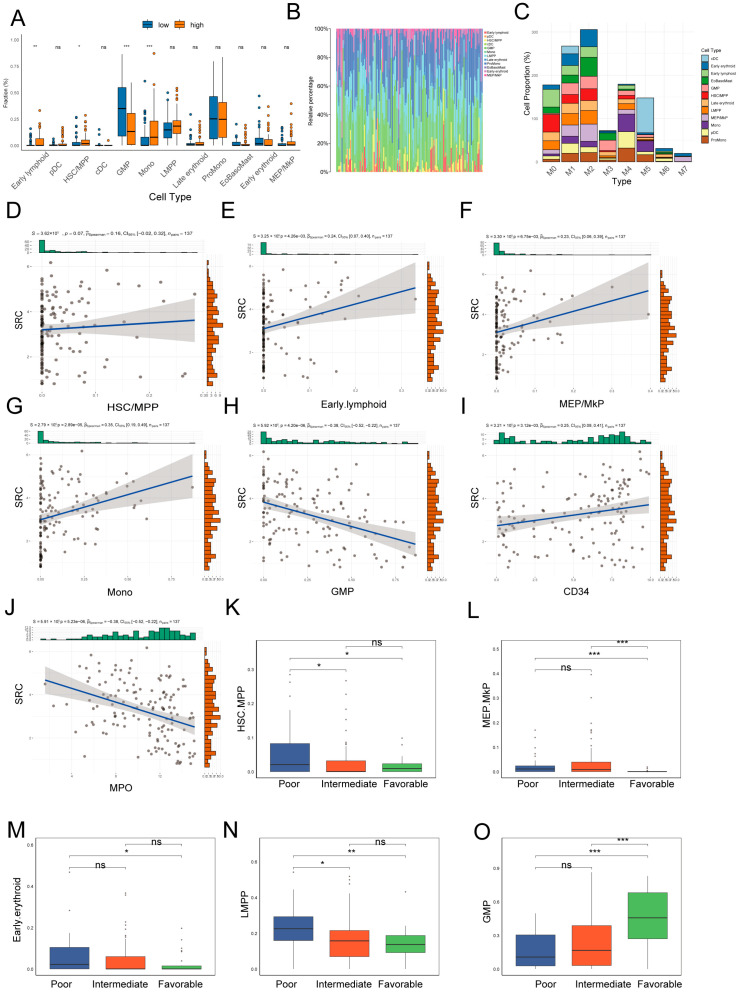
Assessment of tumor microenvironment composition and its clinical associations. (**A**) Comparison of deconvoluted cell proportions by SRC expression. (**B**) Cell type abundance by deconvolution. (**C**) Cell type proportions by FAB group. (**D**–**J**) Evaluating correlations of SRC with cell abundances (**D**–**H**) and key markers (**I**,**J**). (**K**–**O**) Profiling deconvoluted cell proportions based on risk stratification. ns, not significant; * *p* < 0.05; ** *p* < 0.01; *** *p* < 0.001.

To examine the relationship between SRC expression, leukemia cell states, and prognosis, we integrated scRNA-seq-derived signatures with TCGA bulk transcriptomic data. Deconvolution analysis showed that, compared with the SRC-low group, SRC-high samples contained significantly higher proportions of HSCs/MPPs, early lymphoid progenitors, and monocytes (Figure 9A,B). Cell composition patterns across different FAB subtypes are shown in Figure 9C.

Correlation analyses further demonstrated that SRC expression was positively associated with primitive cell states, including HSC/MPP and LMPP, as well as monocytes (Figure 9D–G), and negatively associated with the more differentiated GMP state (Figure 9H). Consistently, SRC expression was positively correlated with primitive-state markers (e.g., CD34) and negatively correlated with differentiation-associated markers (e.g., MPO) (Figure 9I,J).

Importantly, SRC-high samples exhibited a significantly greater proportion of primitive cell states that are associated with poorer prognosis (Figure 9K–N), whereas SRC-low samples were enriched in the relatively more favorable GMP-like state (Figure 9O). Together, these findings support an association between elevated SRC expression, primitive leukemia cell composition, and adverse clinical outcome.

### 2.10. SRC Expression and AML Mutation and Pathway Profiles

**Figure 10:**
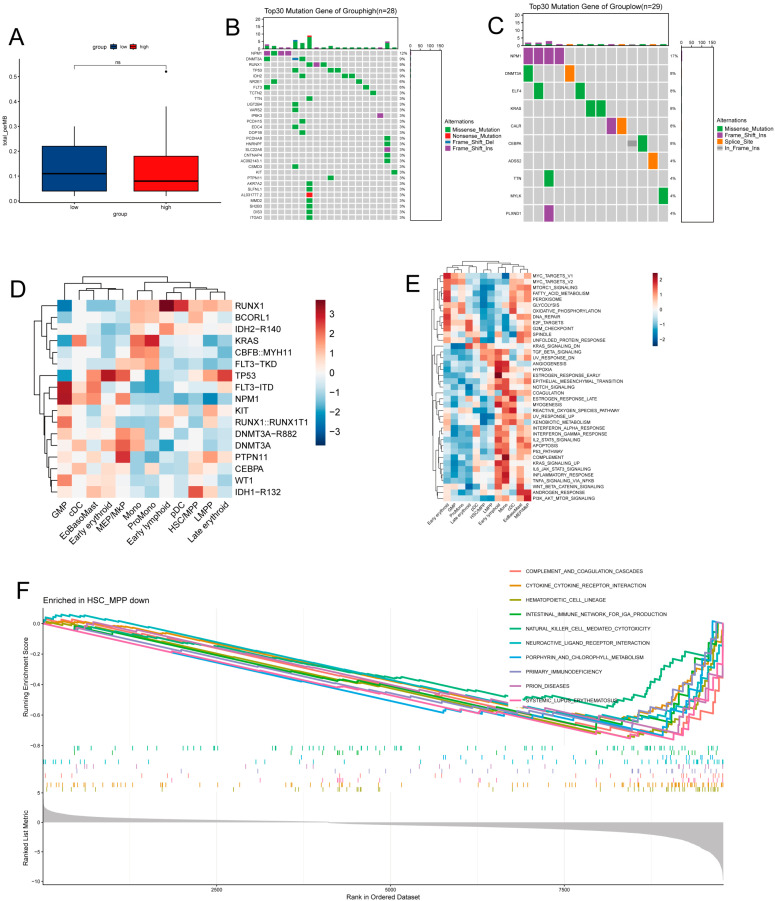
Mutational landscape, signaling heterogeneity, and HSC/MPP enrichment by SRC expression. (**A**) Differential mutation burden between SRC-defined groups. (**B**,**C**) Cohort-specific mutational profiles. (**D**) Distribution of mutations across developmental hierarchies. (**E**) Pathway activity mapped to cellular states. (**F**) Gene set enrichment analysis (GSEA) of HSC/MPP cells.

Mutation analysis showed that the SRC-high group exhibited a distinct mutational landscape characterized by higher frequencies of adverse-risk mutations, including RUNX1 and TP53, whereas the SRC-low group was predominantly enriched for the prognostically favorable NPM1 mutation (Figure 10A–C). At the single-cell level, adverse-risk mutations were mainly concentrated in more primitive cell clusters (e.g., HSC/MPP and LMPP) and certain committed progenitors (e.g., MEP, MkP, and early erythroid cells), while NPM1 mutations were enriched in GMP cells (Figure 10D). These patterns are consistent with the observed association between SRC expression and leukemia differentiation state.

Functional enrichment analysis further indicated that GMP cells preferentially activated cell cycle-related pathways (e.g., E2F targets and MYC targets), reflecting their proliferative characteristics. In contrast, HSC/MPP cells showed negative enrichment for pathways related to hematopoietic lineage commitment, immune response, and cytokine signaling (Figure 10E,F). These state-specific pathway signatures provide a potential molecular framework for understanding cellular heterogeneity and intercellular interactions in the AML microenvironment.

### 2.11. Network Perturbation Analysis Following SRC Knockout

**Figure 11:**
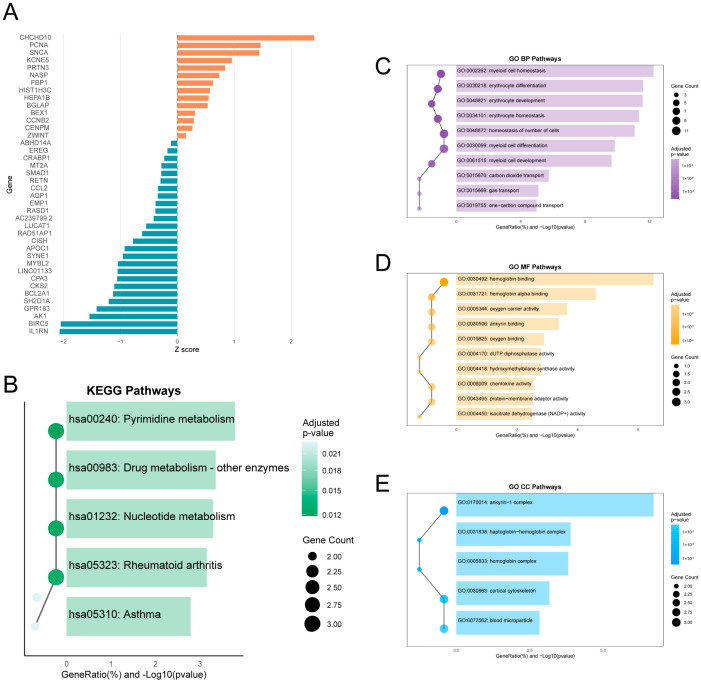
scTenifoldKnk analysis of SRC knockdown in AML. (**A**) Top 40 perturbed genes ranked by |z-score| after SRC knockdown. (**B**) KEGG enrichment of perturbed genes (z-score > 0), showing top pathways. (**C**–**E**) GO enrichment for (**C**) Biological Processes, (**D**) Cellular Components, and (**E**) Molecular Functions. All terms: FDR < 0.05.

To assess the systems-level impact of SRC loss, we performed an in silico SRC knockout analysis using the R package scTenifoldKnk (version 1.0.2). Genes with the highest positive z-scores, indicating the greatest network perturbation following SRC knockout, were selected (Figure 11A) for downstream functional enrichment analysis.

KEGG enrichment analysis showed significant perturbation of multiple metabolic pathways, including pyrimidine metabolism, drug metabolism (other enzymes), and nucleotide metabolism, along with enrichment of immune-related pathways such as rheumatoid arthritis and asthma (Figure 11B). GO enrichment analysis further indicated enrichment in biological processes related to myeloid/erythroid homeostasis and differentiation, cellular components involving hemoglobin- and cytoskeleton-associated complexes, and molecular functions related to oxygen binding and metabolic enzyme activity (Figure 11C–E).

Collectively, these results suggest that SRC perturbation is associated with broad disruption of metabolic, immune-related, and lineage-associated regulatory programs in AML, supporting a central role for SRC in AML network organization and highlighting it as a candidate target for further functional validation.

## 3. Discussion

In the present study, we used an integrative multi-omics strategy combining bulk transcriptomic and single-cell RNA-sequencing data to systematically investigate immune-related molecular determinants in AML and identified SRC as a key hub gene associated with adverse prognosis and microenvironmental remodeling. By integrating WGCNA, PPI network analysis, machine-learning-based feature selection, and survival modeling, we found that SRC was consistently prioritized as a central candidate and was significantly associated with poor overall survival across independent cohorts. In addition, we observed that elevated SRC expression was associated with adverse clinical features, including higher ELN risk categories and unfavorable FAB subtypes. These findings support the clinical relevance of SRC as a prognostic biomarker candidate in AML.

SRC is a non-receptor tyrosine kinase with established roles in oncogenic signaling, cell adhesion, migration, and survival in multiple solid tumors [24,25,26,27,28,29,30]. In hematologic malignancies, SRC-family kinase signaling has also been implicated in leukemogenesis and drug resistance; however, the specific contribution of SRC itself to AML biology, particularly in relation to the immune microenvironment, has remained insufficiently characterized [31,32,33]. Our findings extend previous observations by placing SRC within a broader AML systems-level context, where it appears to be associated not only with leukemic cell-intrinsic states but also with immune and stromal features of the bone marrow niche. This integrative perspective may help explain why SRC-related signaling could have prognostic and therapeutic relevance beyond canonical proliferation pathways.

A notable finding of this study is the robust association between high SRC expression and poor prognosis in both the TCGA-LAML and BeatAML cohorts. Importantly, SRC retained independent prognostic significance in multivariable Cox analysis after adjustment for major clinical covariates, suggesting that its prognostic contribution is not solely a surrogate for established risk factors. We also observed that SRC expression increased across ELN risk strata and varied significantly among FAB subtypes, with lower expression in the favorable M3 subtype and higher expression in poorer-risk subtypes such as M0. Together, these results suggest that SRC expression may reflect underlying disease aggressiveness and differentiation state, and may provide additional value for risk stratification when interpreted alongside conventional clinical and molecular parameters.

Our immune infiltration and pathway analyses suggest that high SRC expression is associated with an immunosuppressive AML microenvironment. Specifically, the SRC-high group showed increased infiltration of M2 macrophages and higher expression of multiple immune checkpoint molecules, including CD274 (PD-L1), CTLA4, LAG3, and TIGIT. In parallel, GSEA demonstrated enrichment of inflammatory and immune-related pathways such as TNF-α/NF-κB signaling, IL6-JAK-STAT3 signaling, and interferon response pathways. These findings support a model in which elevated SRC expression is associated with a leukemia state characterized by immune/microenvironmental remodeling rather than purely proliferative activation. Given that macrophage polarization and checkpoint signaling are both closely linked to immune escape in AML, SRC may represent a molecular feature associated with a more immunoregulatory and therapy-resistant disease context. However, because these observations are based on computational inference from transcriptomic data, the causal role of SRC in shaping these immune phenotypes requires direct experimental validation.

Single-cell analyses provided additional resolution into the potential biological context of SRC expression in AML. We found that SRC expression was enriched in CD34^+^ hematopoietic stem/progenitor compartments, as well as in monocytes and pDCs, and remained relatively sustained along inferred differentiation trajectories from HSCs/MPPs to monocytic states. In contrast to canonical differentiation markers that changed dynamically across pseudotime, SRC showed a broader and more persistent expression pattern. These observations suggest that SRC may be associated with maintenance of signaling competence across primitive and transitional myeloid states, rather than serving as a lineage-restricted differentiation marker. Consistent with this interpretation, deconvolution of bulk RNA-seq data indicated that SRC-high samples were enriched in primitive leukemia-associated cell states (e.g., HSC/MPP- and LMPP-like populations), whereas SRC-low samples were more enriched in relatively differentiated GMP-like states. Because primitive leukemia cell states are commonly linked to treatment resistance and relapse risk, this state association may partly underlie the adverse prognostic impact of SRC-high AML.

Importantly, SRC is widely expressed in various hematopoietic and immune cell subsets. Its expression remains relatively stable along inferred cell differentiation trajectories. This suggests that SRC mainly acts as a microenvironment-related signaling hub, rather than an AML-specific lineage regulator. In this context, SRC can be viewed as a commonly used signaling node. Its clinical prognostic value in AML comes from its involvement in the regulatory programs of high-risk leukemia cell states and the tumor microenvironment. Thus, our results do not imply that SRC is unique to AML biology. Instead, they support that the prognostic and immunological significance of SRC in AML may result from the specific activation and usage of this broad-spectrum signaling molecule in the disease-specific microenvironment.

Our cell–cell communication analyses further suggest that SRC expression is associated with altered signaling architecture within the AML bone marrow microenvironment. Compared with normal bone marrow and SRC-low AML, the SRC-high subgroup showed a reduction in the total number of inferred intercellular interactions, consistent with a communication-constrained or immune-suppressed milieu. At the same time, specific ligand–receptor axes involving MIF-(CD74 + CXCR4), MIF-(CD74 + CD44), and CD99-CD99 appeared selectively enhanced, and SRC expression was positively correlated with key receptor genes involved in these pathways. Moreover, communication pattern analysis suggested a shift in dominant signal recipients from CD8^+^ T cells toward monocytes in the SRC-high subgroup. This pattern may indicate redistribution of leukemia-centered signaling toward myeloid compartments and away from anti-leukemic immune effector interactions. Nonetheless, these findings derive from computational ligand–receptor inference (CellChat) and should be interpreted as hypothesis-generating. Functional perturbation experiments and spatially resolved validation will be important to determine whether SRC directly contributes to these communication changes.

We also observed that SRC-high AML was associated with a distinct mutational landscape, including enrichment of adverse-risk mutations such as RUNX1 and TP53, whereas SRC-low AML was more frequently associated with NPM1 mutation, which is generally linked to more favorable outcomes in specific clinical contexts. At single-cell resolution, adverse-risk mutation signals were concentrated in more primitive cellular compartments, while NPM1 mutation signals were enriched in GMP-like cells. These observations are concordant with the state-distribution analyses and support the possibility that SRC expression may serve as a transcriptomic surrogate of a broader high-risk biological program characterized by primitive cell-state composition, adverse mutation enrichment, and immune microenvironment remodeling. Future studies with matched single-cell genotype–transcriptome profiling would help clarify the extent to which SRC expression is functionally upstream of these phenotypes versus a correlated marker of high-risk AML architecture.

The predicted drug sensitivity analysis provides preliminary therapeutic clues for SRC-high AML. In our transcriptome-based inference, the SRC-high subgroup showed lower predicted IC50 values for several compounds, including afatinib, ibrutinib, and lapatinib. However, these agents are not standard therapies for AML, and the prediction framework used in this study was based on transcriptome-informed pharmacogenomic modeling with the GDSC2 reference dataset rather than AML-specific prospective therapeutic cohorts. Therefore, these findings should be interpreted with caution and regarded as hypothesis-generating only, rather than direct evidence of clinical utility. Further ex vivo and in vivo pharmacologic validation will be required before any translational implication can be considered.

In addition to its potential as a biomarker, SRC may represent a key target with therapeutic relevance in AML. Based on our multi-omics analyses, potential strategies include direct targeting of the SRC/SFK signaling axis, modulation of SRC-linked inflammatory and immune regulatory networks, and combination therapies targeting primitive leukemia cell states and leukemia–microenvironment crosstalk. The observed associations between SRC and immune checkpoint expression, inflammatory activity, and cell–cell communication indicate that SRC-centered interventions may affect both leukemic cell-intrinsic biology and microenvironment-dependent therapeutic sensitivity. As these inferences are generated from computational and transcriptomic analyses, they should be considered hypothesis-generating rather than direct evidence of clinical efficacy, and further pharmacological and mechanistic validation is required to evaluate the translational value of SRC-targeted strategies in AML.

Using scTenifoldKnk, we further performed an in silico perturbation analysis to estimate the systems-level consequences of SRC disruption. The resulting network perturbation signatures suggested broad effects on metabolic pathways, immune-related programs, and lineage-associated regulatory processes. These findings suggest that SRC-centered network perturbation is associated with broad transcriptomic changes in AML-related programs, but they do not by themselves demonstrate that SRC is the primary upstream driver of these processes. Importantly, the virtual knockout analysis provides a computational estimate of network sensitivity rather than direct evidence of gene function under experimental knockout conditions. Nevertheless, it offers a useful framework for prioritizing downstream pathways and candidate readouts for future mechanistic studies. Nevertheless, it offers a useful framework for prioritizing downstream pathways and candidate readouts for future mechanistic studies.

Importantly, the present study does not establish SRC as a causal driver of the immunosuppressive microenvironment, altered differentiation states, or adverse clinical behavior in AML. Because our analyses were largely based on transcriptomic associations, deconvolution, pseudotime inference, and computational network modeling, SRC may also be interpreted as a correlated biomarker or surrogate of a broader high-risk AML program rather than a mechanistically dominant upstream regulator. Thus, although the observed associations are biologically intriguing and internally consistent across multiple analytical layers, they should not be overinterpreted as proof of causality. Future studies using genetic perturbation, gain- and loss-of-function experiments, co-culture systems, and in vivo models will be required to determine whether SRC actively drives these phenotypes or primarily marks aggressive disease states.

This study has several strengths, including the integration of multiple independent bulk and single-cell datasets, external validation of prognostic findings, and the combined use of network analysis, machine-learning-based feature selection, immune deconvolution, and single-cell communication and state analyses to characterize SRC from complementary perspectives. However, several limitations should be acknowledged. This was a retrospective computational study based on public datasets, and key findings such as immune infiltration, ligand–receptor interactions, and drug sensitivity differences were inferred computationally without direct experimental validation. In addition, cohort heterogeneity, batch effects, and differences in sample processing may have influenced some of the single-cell results. Overall, our findings support SRC as an immune-associated and prognostically relevant candidate biomarker in AML, while further mechanistic and prospective clinical validation will be required to clarify its biological and therapeutic relevance.

## 4. Materials and Methods

### 4.1. Data Sources and Preprocessing

This study utilized bulk transcriptomic data and single-cell RNA sequencing (scRNA-seq) data from public databases.

(1) Bulk transcriptomic data: We obtained RNA-seq gene expression data and corresponding clinical information from the TCGA-LAML project within The Cancer Genome Atlas (TCGA) database. After strict quality control (excluding samples with missing survival information or overall survival <30 days), 118 AML samples were included. To increase sample size and validate robustness, the BeatAML validation cohort was also incorporated, comprising 371 samples. Additionally, the GSE9476 dataset from GEO (containing 38 normal donors and 28 AML samples) was used to validate expression differences in the key gene SRC.

(2) Single-cell RNA sequencing data: To analyze single-cell features of the AML bone marrow microenvironment, we used two independent GEO datasets that were analyzed separately rather than jointly integrated. GSE116256 comprises 21 bone marrow samples and 19,115 cells, including 5 healthy donors, 16 diagnostic AML samples, and 19 matched post-treatment AML samples; this dataset was used for non-AML versus AML-related comparisons and corresponding cell–cell communication analyses. GSE289435 comprises 13 AML-related samples and 151,627 cells, including 12 primary AML samples and 1 xenograft-derived sample; because it contains AML-related samples only, it was used for analyses of AML cellular heterogeneity, differentiation trajectories, and leukemia-associated cell states rather than normal-versus-tumor comparisons.

### 4.2. Identification and Functional Enrichment of Immune-Related AML Targets

To identify genes closely associated with the immune microenvironment of AML, we used the XCell algorithm to calculate the ImmuneScore for each sample in the TCGA-LAML cohort. Subsequently, WGCNA was applied for co-expression network analysis. We first preprocessed the gene expression data and selected an appropriate soft thresholding power to construct a stable adjacency matrix and a Topological Overlap Matrix (TOM). Co-expression modules were defined through hierarchical clustering and dynamic tree cutting, with the minimum module gene count set to 120 (minModuleSize = 120). We then calculated the correlation between module eigengenes and ImmuneScore to identify the most relevant key modules. Genes from these key modules were extracted and intersected with AML targets retrieved from the CTD database (http://ctdbase.org/) and the GeneCards database (https://www.genecards.org/) to obtain immune-related acute myeloid leukemia targets. Finally, KEGG and GO enrichment analyses were performed on these targets using the R package clusterProfiler (version 4.18.2).

### 4.3. Core Gene Screening

To identify key genes from the PPI network, we used the Cytoscape cytoHubba plugin (Cytoscape v3.9.1) to further analyze immune-related AML targets. We then combined three different machine learning feature selection algorithms: (1) Least Absolute Shrinkage and Selection Operator (LASSO) regression, employing 10-fold cross-validation and selecting a variable shrinkage model corresponding to the λ.1se criterion; (2) Support Vector Machine–Recursive Feature Elimination (SVM-RFE) algorithm; (3) Random Forest algorithm, assessing variable importance based on the mean decrease in Gini index. Additionally, univariate Cox regression analysis and degree centrality values were used. Finally, SRC was identified as the key hub gene.

### 4.4. Analysis of Clinical and Prognostic Significance of SRC

We focused on the key core gene SRC and conducted an in-depth analysis of its clinical value in AML. By comparing the expression difference of SRC between AML tumor samples and normal bone marrow samples, and utilizing both TCGA and the independent Beat-AML cohort, we assessed the relationship between SRC expression levels and patient overall survival (OS) through Kaplan–Meier survival analysis and Log-rank test. A multivariate Cox proportional hazards regression model was applied to evaluate the independent prognostic impact of gene expression levels. Time-dependent receiver operating characteristic (ROC) curves were plotted, and the area under the curve (AUC) was calculated at specific time points (e.g., 1, 3, and 5 years) to assess the accuracy of gene expression in predicting survival. Furthermore, we analyzed the correlation between SRC expression and clinical characteristics such as European LeukemiaNet (ELN) risk stratification and French–American–British (FAB) classification. Using the R package “rms”, we constructed a nomogram incorporating SRC expression and clinical variables to predict individual patient survival probability. A gene expression-clinical feature association heatmap was also generated.

### 4.5. Tumor Immune Microenvironment and Drug Sensitivity Analysis

Using the xCell algorithm to estimate immune cell infiltration, we compared the composition of the tumor immune microenvironment between the SRC-high and SRC-low expression groups and evaluated differential expression of representative immune checkpoint molecules (e.g., PD-L1, CTLA-4, and LAG-3) between the two groups. In addition, transcriptome-based drug sensitivity prediction was performed using the R package oncoPredict with pharmacogenomic reference data derived from the Genomics of Drug Sensitivity in Cancer version 2 (GDSC2) dataset. Predicted drug response scores/estimated IC50 values for candidate compounds were calculated for TCGA-LAML samples and then compared between the SRC-high and SRC-low groups. These analyses were intended to provide hypothesis-generating therapeutic clues rather than direct evidence of clinical drug response.

### 4.6. Single-Cell RNA Sequencing Data Processing and Analysis

We analyzed the scRNA-seq data using a standard workflow implemented in the Seurat package (v5.0). The pipeline included quality control (retaining cells with 200–6000 detected genes and mitochondrial gene percentage ≤ 10%), data normalization, identification of highly variable genes, and data scaling. Principal component analysis (PCA) was then performed for linear dimensionality reduction. Cell clustering was conducted using the first 30 principal components, with the FindClusters function and a resolution parameter of 1.2. For downstream analyses, we applied UMAP for nonlinear dimensionality reduction and visualization and annotated cell types with reference to the BoneMarrowMap atlas [36]. We further used Monocle2 for pseudotime trajectory inference, AUCell to quantify core gene activity at the single-cell level, CytoTRACE to estimate cellular stemness, and CellChat to infer ligand–receptor interactions and compare intercellular communication networks between groups. The two scRNA-seq datasets were analyzed separately rather than jointly integrated. GSE116256 was used for non-AML versus AML-related comparisons, whereas GSE289435 was used for AML-intrinsic analyses only.

### 4.7. AML Cell Component Deconvolution and Mutation Analysis

To estimate the cellular composition of bulk RNA-seq samples in the TCGA-LAML cohort, we used the CIBERSORTx online platform (https://cibersortx.stanford.edu/, accessed on 15 June 2025). A signature matrix composed of cell subtype-specific gene expression profiles derived from our scRNA-seq data was used as the reference, allowing quantification of the relative proportions of different cell types in each bulk sample.

In parallel, somatic mutation annotation files (MAF format) from TCGA-LAML were processed using the R package maftools, enabling comprehensive summarization, visualization, and comparative analysis of mutation profiles across the whole cohort and among subgroups.

### 4.8. SRC Virtual Knockout Analysis

To simulate the transcriptional impact of SRC knockout on the gene regulatory network, we applied the scTenifoldKnk algorithm [37]. This method first constructs a gene regulatory network using wild-type scRNA-seq data. A virtual knockout network is then generated by setting the edge weights associated with SRC to zero. The wild-type and knockout networks are subsequently compared using manifold alignment, and the projection distance (d-value) of each gene in the low-dimensional space is calculated to quantify its positional shift within the network. To assess statistical significance, a null distribution was generated using 50 random samplings, and the observed d-values were converted to z-scores. KEGG and GO enrichment analyses were then performed on significantly perturbed genes (Z-score > 0, *p* < 0.05, log2FC > 2) using the clusterProfiler package to infer potential downstream regulatory functions of SRC.

### 4.9. Statistics and Software

Unless otherwise specified, continuous variables were compared using the Wilcoxon rank-sum test, and categorical variables were compared using the chi-square test. Correlation analyses were performed using Spearman’s rank correlation. All statistical analyses were conducted in the R environment (version 4.5.2). A two-sided *p* value < 0.05 was considered statistically significant.

## 5. Conclusions

Using an integrative framework combining bulk and single-cell transcriptomic analyses, we identified SRC as an immune-related hub gene associated with adverse prognosis in AML. High SRC expression was linked to unfavorable clinical features, primitive leukemia-associated cell states, immunosuppressive microenvironmental characteristics, and distinct mutational/pathway profiles. Computational drug-response prediction and in silico network perturbation analyses further suggested potential biological relevance of SRC-centered programs, although their translational implications require substantial further validation. Overall, these findings support SRC as a candidate biomarker for risk stratification in AML, while suggesting that its clinical relevance may reflect disease-context-dependent deployment of a broadly active signaling hub rather than a uniquely AML-specific regulator.

## Data Availability

The data presented in this study are available in public repositories. These data were derived from the following resources available in the public domain: The Cancer Genome Atlas (TCGA)-Acute Myeloid Leukemia (LAML) project (https://portal.gdc.cancer.gov/, accessed on 15 June 2025); BeatAML cohort (http://www.vizome.org/, accessed on 15 June 2025); Gene Expression Omnibus (GEO)-GSE9476, GSE289435, GSE116256 (https://www.ncbi.nlm.nih.gov/geo/, accessed on 1 July 2025); CTD (Comparative Toxicogenomics Database) (http://ctdbase.org/); GeneCards (https://www.genecards.org/); Cancer Cell Line Encyclopedia (CCLE) (https://sites.broadinstitute.org/ccle/, accessed on 1 July 2025). Custom analysis scripts and processed data generated during this study are available from the corresponding author upon reasonable request.

## Abbreviations

The following abbreviations are used in this manuscript:

AML	Acute Myeloid Leukemia
AUC	Area Under the Curve
BCL-2	B-cell Lymphoma 2
BP	Biological Process
CC	Cellular Component
CCLE	Cancer Cell Line Encyclopedia
CTD	Comparative Toxicogenomics Database
CTLA-4	Cytotoxic T-Lymphocyte Associated Protein 4
ELN	European LeukemiaNet
FAB	French–American–British
FAK	Focal Adhesion Kinase
FLT3	FMS-like Tyrosine Kinase 3
GEO	Gene Expression Omnibus
GO	Gene Ontology
GMP	Granulocyte-Monocyte Progenitor
GSEA	Gene Set Enrichment Analysis
HSC	Hematopoietic Stem Cell
HSPC	Hematopoietic Stem/Progenitor Cell
IC50	Half Maximal Inhibitory Concentration
IDH	Isocitrate Dehydrogenase
IRAT	Immune-Related AML Target
KEGG	Kyoto Encyclopedia of Genes and Genomes
LAG-3	Lymphocyte Activation Gene-3
LASSO	Least Absolute Shrinkage and Selection Operator
LMPP	Lymphoid-primed Multipotent Progenitor
LSC	Leukemia Stem Cell
MAF	Mutation Annotation Format
MCL-1	Myeloid Cell Leukemia 1
MEP	Megakaryocyte-Erythroid Progenitor
MF	Molecular Function
MIF	Macrophage Migration Inhibitory Factor
MM	Module Membership
MPO	Myeloperoxidase
MPP	Multipotent Progenitor
NF-κB	Nuclear Factor kappa-B
NK cell	Natural Killer cell
NPM1	Nucleophosmin 1
OS	Overall Survival
PCA	Principal Component Analysis
pDC	Plasmacytoid Dendritic Cell
PD-L1	Programmed Death-Ligand 1
PPI	Protein–Protein Interaction
ROC	Receiver Operating Characteristic
RUNX1	Runt-related Transcription Factor 1
scRNA-seq	Single-cell RNA Sequencing
SRC	Sarcoma (Proto-oncogene)
SVM-RFE	Support Vector Machine-Recursive Feature Elimination
TCGA	The Cancer Genome Atlas
TIGIT	T-cell Immunoreceptor with Ig and ITIM domains
TME	Tumor Microenvironment
TNF-α	Tumor Necrosis Factor-alpha
TOM	Topological Overlap Matrix
TP53	Tumor Protein p53
UMAP	Uniform Manifold Approximation and Projection
VEGF	Vascular Endothelial Growth Factor
WGCNA	Weighted Gene Co-expression Network Analysis
